# Water Quality, Sanitation, and Hygiene Conditions in Schools and Households in Dolakha and Ramechhap Districts, Nepal: Results from A Cross-Sectional Survey

**DOI:** 10.3390/ijerph14010089

**Published:** 2017-01-18

**Authors:** Akina Shrestha, Subodh Sharma, Jana Gerold, Séverine Erismann, Sanjay Sagar, Rajendra Koju, Christian Schindler, Peter Odermatt, Jürg Utzinger, Guéladio Cissé

**Affiliations:** 1 Swiss Tropical and Public Health Institute, Department of Epidemiology and Public Health, P.O. Box, CH-4002 Basel, Switzerland; akina.shrestha@unibas.ch (A.S.); jana.gerold@unibas.ch (J.G.); severine.erismann@unibas.ch (S.E.); sanjay.sagar@unibas.ch (S.S.); christian.schindler@unibas.ch (C.S.); peter.odermatt@unibas.ch (P.O.); juerg.utzinger@unibas.ch (J.U.); 2 University of Basel, Petersplatz 1, CH-4001 Basel, Switzerland; 3 School of Medical Sciences, Kathmandu University, P.O. Box 11008, Dhulikhel, Nepal; rajendrakoju@gmail.com; 4 Aquatic Ecology Centre, School of Science, Kathmandu University, P.O. Box 6250, Dhulikhel, Nepal; subodh.sharma@ku.edu.np

**Keywords:** cross-sectional survey, drinking water quality, hygiene, Nepal, sanitation, schoolchildren

## Abstract

This study assessed drinking water quality, sanitation, and hygiene (WASH) conditions among 708 schoolchildren and 562 households in Dolakha and Ramechhap districts of Nepal. Cross-sectional surveys were carried out in March and June 2015. A Delagua water quality testing kit was employed on 634 water samples obtained from 16 purposively selected schools, 40 community water sources, and 562 households to examine water quality. A flame atomic absorption spectrophotometer was used to test lead and arsenic content of the same samples. Additionally, a questionnaire survey was conducted to obtain WASH predictors. A total of 75% of school drinking water source samples and 76.9% point-of-use samples (water bottles) at schools, 39.5% water source samples in the community, and 27.4% point-of-use samples at household levels were contaminated with thermo-tolerant coliforms. The values of water samples for pH (6.8–7.6), free and total residual chlorine (0.1–0.5 mg/L), mean lead concentration (0.01 mg/L), and mean arsenic concentration (0.05 mg/L) were within national drinking water quality standards. The presence of domestic animals roaming inside schoolchildren’s homes was significantly associated with drinking water contamination (adjusted odds ratio: 1.64; 95% confidence interval: 1.08–2.50; *p* = 0.02). Our findings call for an improvement of WASH conditions at the unit of school, households, and communities.

## 1. Introduction

Water, sanitation, and hygiene (WASH) are fundamental to human development and wellbeing. The World Health Organization /United Nations Children’s Fund (WHO/UNICEF) Joint Monitoring Program (JMP) for water supply and sanitation estimates that, in 2015, 663 million people lacked improved drinking water sources and 2.4 billion lacked improved sanitation facilities [1]. Unsafe and insufficient quantity of drinking water, inadequate sanitation, and unimproved hygiene account for 7% of the global burden of disease and 19% of child mortality worldwide [2,3]. The era of the Millennium Development Goals (MDGs) from 2000–2015 had specific targets for “improved” access to drinking water supply and “basic sanitation”; however, coverage fell short of the sanitation target [1,4]. Still today, many schools and households in low- and middle-income countries (LMICs) lack adequate and safe WASH services, compromising people’s health and wellbeing [5]. For example, in 2012, UNICEF reported that only 51% of schools in LMICs, had access to adequate water and 45% had access to sanitation facilities [6]. The lack of reliable access to safe and sustainable WASH infrastructure, in conjugation with related hygiene and sanitation behaviours, remains a major public health problem [7,8,9,10,11]. In LMICs, each year, 1.5–2 million children die from WASH-related diseases and many more are debilitated by illness, pain, and discomfort [12]. While the majority of deaths occur in children below the age of five years, the burden of disease among schoolchildren is considerable [13]. Approximately 74% of the health burden in schoolchildren in LMICs is due to intestinal helminth infections and 60% of the mortality is linked to infectious diseases such as schistosomiasis, soil-transmitted helminthiasis, and trachoma [14]. Approximately 88% of diarrhoeal diseases and 47% of soil-transmitted helminthiasis are due to WASH issues in LMICs, which in turn cause malnutrition and impair food intake and nutrient absorption [10,12,15,16].

The heavy metals such as arsenic and lead introduced into drinking water primarily by dissolution of naturally occurring ores, minerals, and industrial effluents are public health problems. Arsenic is one of the most dangerous trace elements and is predominantly found in rocks, soils, and natural water. The studies reported that arsenic affects the organs and systems in the body, including skin, heart, respiratory organs, and kidney consequently leading to cancer of the lung, kidney, and bladder [17]. Similarly, lead, another heavy metal, acts as an anti-essential trace element, highly toxic cumulative element in the human body and is widely distributed in soil and groundwater [18,19]. For neurological, metabolic, and behavioural reasons, children are more vulnerable to the effects of lead compared to adults [20].

Nepal faces a plethora of problems related to WASH issues [21,22]. In 2015, the World Health WHO/UNICEF JMP reported that 92% of the Nepalese population had access to improved water, and hence, met this specific MDG target [1]. However, it remains to be determined whether the water classified as improved is safe for consumption. Sanitation coverage was 46%, while 37% of the population were still practicing open defecation, causing serious risks of environmental contamination, such as to open water sources [1,23]. At the unit of the school, 61.9% had at least one toilet facility. Water supply facilities are not adequate to meet and maintain sanitation requirements in most of the schools [24]. According to data from the Department of Health Service in Nepal, about 3500 children die each year due to water-borne diseases [25]. Intestinal parasitic infections and diarrhoeal diseases due to inadequate WASH are the principal causes [26,27]. The most common intestinal helminths among Nepalese children reported are *Ascaris lumbricoides*, hookworm, and *Trichuris trichiura*, with manifestations that include malnutrition, iron deficiency anaemia, malabsorption syndrome, intestinal obstruction, and impaired physical growth [27]. There is a large body of evidence indicating that WASH interventions improve health and lead to significant reductions in both the severity and prevalence of diarrhoea and helminthiases [5,28,29]. Several studies investigated heavy metals, such as lead and arsenic. With regard to lead, a study reported high concentrations (15–35 µg/L) in drinking water samples collected from different parts of Nepal [30]. Meanwhile, a study investigating the quality of groundwater, especially in the Terai region, revealed high arsenic content [31]. Furthermore, some studies have revealed high concentrations of arsenic in shallow tube wells (<50 m depth) with reported arsenic concentrations of up to 10 µg/L [32,33].

The project entitled “Vegetables go to School: improving nutrition through agricultural diversification” (VgtS) is a multi-country study that seeks to deepen the understanding of whether school vegetable gardens, nutrition, and WASH interventions might lower the incidence of intestinal parasitic infections among schoolchildren and reduce malnutrition. Five countries are involved: Bhutan, Burkina Faso, Indonesia, Nepal, and the Philippines. The study protocol for Burkina Faso and Nepal has been published elsewhere [34]. The specific objectives of the research presented here were: (i) to assess WASH conditions at the units of the school, households, and community; (ii) to conduct a baseline appraisal and identify gaps from which to identify priority needs and required interventions; and (iii) to analyse the association between water contamination and WASH predictors at the household level. We examined the water quality (physiochemical characteristics, microbiological contamination by thermo-tolerant coliforms (TTC), and heavy metals content), and sanitation and hygiene conditions at schools, households, and communities of the sampled children.

## 2. Materials and Methods

### 2.1. Study Sites

**Figure 1:**
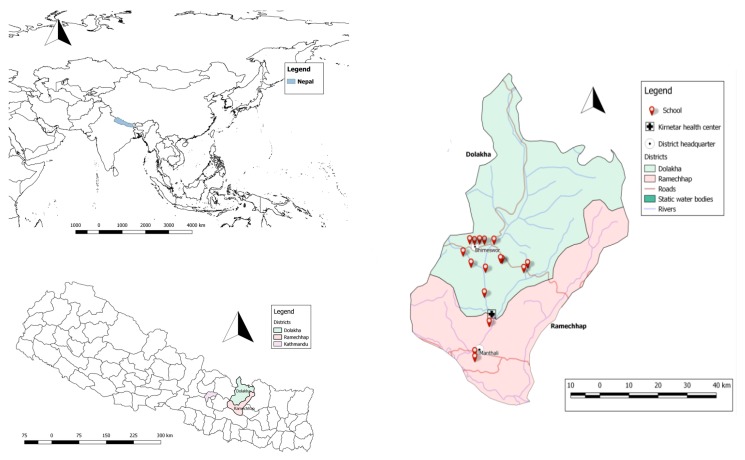
**Top left**: World map showing Nepal; **Bottom left**: Map of Nepal highlighting Dolakha and Ramechhap districts; **Right**: Map of Dolakha and Ramechhap districts showing the surveyed schools.

This study was conducted at the VgtS project sites in Dolakha and Ramechhap districts in Nepal. Dolakha is located approximately 180 km and Ramechhap approximately 150 km from Kathmandu, the capital of Nepal (Figure 1). Most of Dolakha district lies in the temperate zone (28.5%), followed by subtropical (26.2%), nival (17.4%), subalpine (16.6%), alpine (9.4%), and tropical (1.9%) zones. Similarly, Ramechhap district lies mostly in the subtropical zone (42.1%), followed by temperate (21.0%), tropical (18.0%), nival (7.3%), subalpine (6.7%), and alpine (3.6%) zones [35]. Dolakha and Ramechhap districts have 54 village development committees (VDCs) and one municipality. We collected water samples from 32 VDCs in Dolakha and eight in Ramechhap.

### 2.2. School Selection

In mid-2015, a cross-sectional survey was conducted in 16 schools (13 in Dolakha and three in Ramechhap) of the 30 purposively selected schools of the National Agriculture Research Council and the Department of Education, within the frame of the VgtS project (Figure 1). Selection criteria were as follows: (i) schools located within 1-h walking distance from a main street; and (ii) water available at school for vegetable cultivation. In the Ramechhap district, only three schools were selected, as the aforementioned criteria were hard to meet.

The 16 schools were randomised to one of four interventions, as follows:

- schools benefiting from a school garden intervention only;
- schools allocated a nutrition and health education programme, including school garden implementation;
- schools benefiting from nutrition and WASH intervention; and
- schools without any interventions (considered as control schools).

### 2.3. Study Population and Sample Size

The study population consisted of children in grades 6 and 7, aged 8–16 years. A Monte Carlo simulation showed that 800 children, with 50 children per school and four schools per intervention arm would provide at least 75% power for finding simultaneous significant effects of the implemented type of intervention under the following assumptions:

- the prevalence of intestinal protozoan and helminth infections is about 30% [36] and remains constant in the absence of any intervention;
- the probability of new intestinal protozoa and helminth infections at the end of follow-up is 15%;
- the same effect odds ratios (ORs) apply to incidence and persistence of intestinal protozoa and helminth infection; and
- each of the two interventions reduces the odds of infection by 50%, and their effects are additive on the logit-scale.

### 2.4. Sample Collection and Treatment

Sterilised polyethylene bottles (250 mL) were used for water sampling. Membrane filter and membrane lauryl sulphate broth were used in the estimation of TTC. Water samples were collected from:

- schools, between 30 May and 6 June 2015, from one main functioning drinking water point in each of 16 schools and 13 point-of-use (water container, cups);
- households, between 6 and 30 June 2015, at point-of-use in every 562 surveyed schoolchildren’s households; and
- communities, from 1 to 10 June 2015, in approximately 10% of the water sources in the community. Of note, 43 drinking water sources were collected from 40 communities selected at random (at least one sample per community). Water was collected from stand pipes (*n* = 37), protected springs (*n* = 3), protected wells (*n* = 2), and ponds (*n* = 1).

The sample collections were done from the stand pipes and springs, ponds, wells, and reservoirs according to the standard guidelines of the Delagua water testing kit. To collect water samples from stand pipes, the tap was opened for 1 min before taking a sample. This ensured that any deposits in the pipes were washed out and the water sample was representative of the water in the supply pipes. To collect water from ponds, reservoirs, open wells, or other surface water sources, the sterilized cups were rinsed twice with the specific water source before taking the sample [37].

### 2.5. Physical, Chemical, and Microbiological Parameters

Physical parameters of the water sample were measured, including temperature (°C), pH, and turbidity (nephelometric turbidity unit (NTU)). Similarly, chemical parameters were measured, such as residual chlorine (free and total), lead, and arsenic contents. Measured microbiological parameters included TTC. The standards of each parameter are 5 NTU for turbidity, 6.5–8.5 for pH, 0.01 mg/L for lead, 0.05 mg/L for arsenic, 0.1–0.2 mg/L for residual chlorine, and <1 for TTC as per the national drinking water quality standard guideline (NDWQS) of the Government of Nepal [38].

### 2.6. Drinking Water Quality Analysis

Drinking water samples were collected according to the standard guidelines of the Delagua water testing kit [37]. The 250 mL polyethylene bottles were sterilised in an autoclave at 121 °C for 15 min. These sample bottles were then rinsed three times by the water collected for analysis, made watertight by air tightening and marked with a unique code and date of sampling. The water samples were stored in a portable cool box, transferred to the laboratory within 3 h of collection, and stored at 4 °C in a refrigerator preceding analysis done within a maximum of 30 h. The water samples were brought to room temperature before analysis. We filtered 100 mL of each sample using sterile filter paper with a 0.45 μm pore size, applied vacuum suction, and incubated at 44 °C for 18 h. After incubation, bacteria were enumerated by colony count [37].

### 2.7. Heavy Metal Analysis

Lead and arsenic contents were analysed in all 16 samples from the schools. The samples were subjected to a flame atomic absorption spectrophotometer (AAS, model 2380, Perkin-Elmer GmbH, Überlingen, Germany); in combination with high-pressure liquid chromatography (HPLC, Akvilon, Moscow, Russia) for arsenic. Standardisation of the instrument was carried out before laboratory procedures to verify consistency in instrument response. In each water sample, lead and arsenic contents were determined in triplicate for quality control.

### 2.8. Questionnaire Survey

A semi-structured questionnaire was used to determine WASH conditions for schools, surveyed schoolchildren, and their households. School WASH information was obtained from the school principals. Observational measures were used to collect information related to the cleanliness of the latrine and availability of water around-the-clock. Knowledge, attitude, and practices (KAP) related to WASH were collected from schoolchildren. Household-related WASH information was collected from caregivers. Questions included topics such as availability of improved water in households, water treatment, livestock, and disease prevalence in the preceding two weeks and socio-demographic information. Data were collected using tablets (Samsung Galaxy note 10.1 N8010, Seoul, Korea) and open data kit (ODK) software (University of Washington, Seattle WA, USA). To ensure the reliability of the information, schoolchildren and their caregivers were interviewed in their mother tongue by enumerators familiar with the study area and fluent in local languages. The data collection device was password-protected and automatic deletion of data after synchronising with the server was activated to maintain confidentiality. The data were thereafter transferred and stored electronically in a password-protected server at the Swiss Tropical and Public Health Institute (Swiss TPH, Basel, Switzerland). Analysis was done using STATA version 14 (Stata Corporation, College Station, TX, USA).

### 2.9. Statistical Analysis

Water quality data were entered into an Excel 2010 spread sheet (Microsoft, Redmond, WA, USA). A new variable for socioeconomic status was created using factor analysis of 13 asset indicator variables and retaining the first factor. The households were then classified into one of three categories: high, middle, and poor socioeconomic status, using the k-means procedure. New variables for sanitation and hygiene in school were also created using factor analysis of respective indicator variables, and we retained the first factor of each analysis. Both factor scores were classified into two categories—adequate and inadequate, using the k-means procedure. Similarly, a new variable for hygiene for the schoolchildren and their caregivers was created using factor analysis separately with two conceptually similar binary variables of mode of hand-washing (with water only, ash, mud/soil, water and soap, no hand washing); and its occasions (for schoolchildren: before eating, after eating, after playing, after toilet; and for their caregivers: before preparing food, before eating, after eating, after defecation, after child’s defecation, before breastfeeding, after breastfeeding, no hand-washing). The score of the first factor was then classified into three categories: high, middle, and low using the k-means procedure. Mixed logistic regression adjusted for the clustering of data within schools was applied to investigate the association between the dependent variable; namely, TTC and 14 independent variables (e.g., household drinking water and water treatment) based on a literature review [39,40]. The outcome variable (i.e., TTC) was treated as binary outcome representing absence (TTC = 0) or presence (TTC = 1) of TTC in the water sample, the latter category also applying if TTC were too numerous to be counted (i.e., >100). ORs were calculated, including 95% confidence intervals (CI) and Wald test *p*-values were obtained. Explanatory variables in the final mixed logistic regression model included household drinking water source (i.e., private tap, shared tap, public tap, and other sources), container for fetching water (i.e., clay pot, plastic container, and metal container), and livestock kept inside the house. Adjustments were made for potential confounder variables, such as regional differences, educational attainment of the caregivers, and socioeconomic status. All variables were assessed one-by-one and retained for the maximal model if their *p*-value was <0.2. Backward stepwise elimination was used in the multivariable logistic regression with school as a random effect and removing non-predicting covariates up to a significance level of 0.2. Associations were considered statistically significant if *p*-values were <0.05. The results of physicochemical and microbiological analyses were compared with the NDWQG by the Government of Nepal (Table 1) [38].

### 2.10. Ethics Statement

The study was approved by the research commission of Swiss TPH (FK No. 116; approval date: 30 October 2014). Ethical approval was obtained from the “Ethikkommission Nordwest- und Zentralschweiz” (EKNZ) in Switzerland (reference number UBE-15/02; approval date: 12 January 2015), the institutional review board of Kathmandu University, School of Medical Sciences, Dhulikhel Hospital, Nepal (reference No. 86/14; approval date: 24 August 2014), and the institutional review board, Nepal Health Research Council (reference No 565; approval date: 11 November 2014). The study is registered at International Standard Randomised Controlled Trial Number register (identifier: ISRCTN30840; date assigned: 17 July 2015). The schools and households with TTC were provided with chlorine solution and health promotion programmes. Community stakeholders were informed about the status of water sources in their communities.

## 3. Results

### 3.1. Study Compliance and Population Characteristics

A total of 708 children were included in the study. However, due to an earthquake that hit in the midst of the study period and damaged most of the houses, 146 caregivers were not accessible. Complete data were available from 708 schoolchildren and 562 households, and were used for final analysis.

The socio-demographic characteristics of the schoolchildren and their caregivers are summarised in Table 1. There were similar numbers of boys and girls participating in the 16 schools. The median age of the children was 13 years with an interquartile range of 2 years. Over one-third of caregivers did not have any formal education. Three-quarters of the houses of schoolchildren were made up of iron sheets for walls and roofs and mud for floors. Domestic animals were kept by over 90% of the households, while over one-third of caregivers reported to have animals freely roaming inside their houses.

### 3.2. School and Community WASH Characteristics

Table 2 summarises school condition and WASH characteristics at the unit of the school. About one-third of schools were constructed more than 20 years ago with three-quarters of the schools having at least 500 pupils. About three-quarters of the schools were built with iron sheets (walls and roofs) and mud (floor). Fourteen out of 16 schools had some type of water infrastructure (standpipe and piped water into the dwelling); however, several were broken at the time of the survey. None of the schools had round-the-clock availability of water. Only around one-third of the schools reported having drinking water available throughout the year. None of the school principals reported that water at the school was treated prior to consumption. Drinking water quality testing by a health inspection team within two months prior to the survey occurred in only one of the 16 schools surveyed.

Table 3 summarises the physicochemical and microbiological parameters of the water samples taken from schools and communities, including turbidity, pH, chlorine, and TTC. The average temperature of water samples obtained in schools of Dolakha and Ramechhap districts was 13.2 °C and 16.0 °C, respectively. The median turbidity was 2.5 NTU (range: 0–5 NTU). The median turbidity of school point-of-use water samples was 10.1 NTU (range: 5–20 NTU). The average turbidity of school point-of-use water samples was 6.15 NTU. In the communities, four out of 43 water sources had a turbidity >5 NTU, while turbidity of the remaining 39 sources were between 2 NTU and 5 NTU. The pH level of functioning school water points had an average of 6.9 (range: 6.8–7.6). The average pH of point-of-use water samples was 7.0 with a range between 6.8 and 7.4. Similarly, for the community water points, the average pH was 6.8 with a range between 6.8 and 7.2.

The median of free and total chlorine of the main drinking water points and the point-of-use water samples from schools were 0.1 mg/L and 0.5 mg/L, respectively. The free residual chlorine of the water sources in the communities were within the acceptable national limits (0.1–0.2 mg/L). All the water samples from the communities had total residual chlorine below 1 mg/L. The lead and arsenic levels in school drinking water sources were, on average, 0.01 mg/L and 0.05 mg/L, respectively, hence below the national standards.

Of the 16 school drinking water sources, 6.2% had TTC levels >100 colony forming unit (CFU) per 100 mL. The median value of TTC was 6 CFU/100 mL. Of the 13 water samples obtained from school points-of-use, 30.8% had >100 CFU/100 mL. Of the 43 community water source samples, 18.6% ranged between 11 and 100 CFU/100 mL and 13.9% had values of 100 CFU/100 mL and above.

In the study area, more than half of the schools had a pit latrine without cement floor. Although water seal latrines on the premises were seen, 41.6% of the latrines were not used as they were full. None of the schools had toilet paper and only 12.5% of the schools had regular water supply for anal cleansing. In all schools where latrines were present, the use was separated by gender. In schools with at least one toilet, the median student-to-toilet ratio was 65 per toilet. Approximately 4% of children reported that they did not use the latrine at school. Only 6.3% of schools reported that they cleaned the latrine each week; the rest reported less frequent cleaning. None of the schools had a dedicated budget for purchasing cleaning supplies for the latrines or soap for handwashing. The survey also investigated the implementation of any hygiene training programme at school during the past two months. More than half of the schools (62.5%) had never implemented a hygiene programme, including a hygiene component and school action plan. Furthermore, handwashing stations and soap were not available at any of the surveyed schools.

### 3.3. KAP of Schoolchildren on WASH at Schools and Households

The findings from the KAP survey pertaining to WASH among schoolchildren are shown in Table 4. More than half of the schoolchildren reported washing their hands with soap and water before eating and after defecation. However, 11.7% reported that they did not wash their hands at any of these occasions. Over 90% of schoolchildren reported that they regularly drank water at the school. Around 90% of schoolchildren reported that they had heard about dirty water causing illness, however, they were not aware about specific types of water-borne diseases and modes of transmission.

### 3.4. WASH Characteristics of Households

WASH information for schoolchildren’s households is also summarised in Table 4. Water sources were categorised as improved (i.e., private tap, shared tap, public tap, hand pump, protected deep well, bore hole, and protected springs) and unimproved (i.e., non-improved source, including surface water such as river, lake swamp, and ponds). While 44.7% of households did not have a piped water distribution network, 78.1% reported having insufficient drinking water throughout the year. A total of 22.6% households did not cover their water container and 86.4% of households did not treat drinking water. Among those households that treated water, boiling was the most commonly known means of purification (36.1%).

Table 5 summarises the physicochemical and microbiological parameters of the water samples taken from the households. The average turbidity recorded in the household point-of-use water samples was 6.4 NTU (range: 5–14 NTU); average pH was 6.9 (range: 6.5–7.6); the average free chlorine was 0.14 mg/L (range: 0.1–0.3 mg/L), and the average total chlorine was 0.5 mg/L (range: 0.1–1.0 mg/L). Drinking water was contaminated with TTC in 27.4% of the household point-of-use water samples. Out of 562 water samples examined, 12.5% had >100 CFU/100 mL. 

Almost half (45.7%) of the caregivers reported that children had suffered from water-borne diseases within the two weeks preceding the survey. A total of 38.2% of caregivers complained of dysentery, 30.9% of fever, 22.4% of watery diarrhoea, while 8.5% reported other conditions of ill-health.

### 3.5. Association of TTC with Household WASH Predictors

Table 6 shows the association of water contaminated with TTC with household WASH predictors. Significant differences in TTC were observed between the two districts, with Ramechhap having higher odds of TTC compared to Dolakha district (adjusted odds ratio (aOR) 2.25, 95% CI: 1.16–4.34). We found a significant association between domestic animals freely roaming in households and contamination of water with TTC compared to household without freely roaming domestic animals (aOR 1.64, 95% CI: 1.08–2.50). Households using a protected spring water source for drinking were more likely to experience TTC contamination, but the association lacked statistical significance (aOR 2.48, 95% CI: 0.64–9.66).

## 4. Discussion

Our study revealed several WASH challenges at the unit of the school, household, and community in the districts of Dolakha and Ramechhap in Nepal. Indeed, our data provide evidence of inadequate drinking water availability at the main water sources in the schools surveyed. Moreover, water samples subjected to chemical and microbial tests revealed considerable faecal contamination. The access to “safe” water coverage from improved water sources in 12 schools was, in fact, not safe for consumption. Contamination of water samples with >100 TTC CFU/100 mL was detected in about one-third of the water samples obtained from schools. Furthermore, due to inadequate availability of drinking water at 14 schools, children obtain drinking water from other locations where safe drinking water consumption is not guaranteed.

Linking observational WASH assessment, out of all the surveyed schools, more than a quarter of schools had no sanitation infrastructures with a regular water supply available for anal cleansing. The conditions of latrines were poor and lacked essential hygiene materials (e.g., soap). Moreover, none of the schools had separate handwashing stations in close proximity to the sanitation infrastructure for handwashing. Additionally, none of the schools had any allocated budget for purchasing toilet-cleansing supplies. Another challenge identified by our study is insufficient coverage of improved/sanitary latrines and handwashing stations within the schools. The majority of schools did not meet the national student-to-toilet standard set by the Government of Nepal where one latrine per 50 students and at least one set of handwashing stations for a set of latrines (one for boys, one for girls) were recommended [24]. To improve this ratio, the school committee or parents’ associations might focus their efforts also on building an adequate number of toilets for girls and boys. In addition to latrines, building more urinals for boys (which have considerably lower costs than latrines) could also be beneficial for schoolchildren. We found that the surveyed schools had usually one or two water taps available at a school for handwashing, and these were located at central places, far away from latrines. The findings of this study regarding WASH in schools are consistent with evidence on WASH in schools in Nicaragua where schools were without adequate sanitation infrastructures and handwashing facilities, highlighting several WASH challenges [39]. Similar observations have been made in South Africa where the majority of the schools had access to unhygienic pit latrine and had one water tap, which was mostly located at a central point on the school premises [41].

In terms of WASH at the unit of household, more than half of the households had access to an improved water source and sanitary infrastructure. However, water quality was typically not suitable for drinking in 112 households (20.0%). The water qualities from stored household samples were found to be worse than the water samples from the community source. This might be due to further contamination during transportation, storage, and point-of-use at households. This finding was consistent with the evidence from meta-analysis that reported the association of supply type with faecal contamination of source of water and household stored drinking water in LMICs [29,40].

In the case of water quality of the samples obtained from the community, more than 30% were contaminated by TTC with maximum coliform count of >100 CFU/100 mL, with drinking water quality standards exceeded in 14 (32.5%) water sources. This finding is consistent with a prior study conducted in the communities of Kathmandu valley and Myagdi district of Nepal, where a maximum TTC of 267 CFU/100 mL had been reported [42] and where 27.3% water sources were contaminated with TTC, respectively [17].

Our survey included the examination of the physicochemical quality of water samples. Importantly, most of the physicochemical parameters were within national thresholds, except for turbidity. Some of the water samples showed high turbidity (>10 NTU), which might be due to the discharge of domestic effluents and runoff from agricultural activities. In turn, this might call for adequate and proper treatment of water before consumption [40]. The pH was within the national standard (6.5–8.5). The schools, households, and communities mostly had a natural water source, and hence, pH levels were expected to be in this range. Similar observations have been reported from studies conducted in Myagdi district and Dharan, where pH levels of 7.6 were reported [17,43].

When drinking water leaves a water point (e.g., tap), a residual free chlorine of about 1 mg/L is recommended, and similar levels are recommended for points-of-use during consumption [44]. In our study, none of the stored water samples from schools and households had detectable residual free chlorine of 1 mg/L, even though chlorine solution had been distributed free of charge by various relief organisations after the April 2015 earthquake. The possible explanation for the low levels of detectable residual free chlorine might be that the aftershocks due to the earthquake were still quite frequent during the survey period, and hence, the chlorine promotion programme might have received only little attention, or people may dislike the odour of chlorine or they might regard chlorination as being an extra form of work during an emergency period.

Regarding heavy metals, fortunately, our investigation revealed acceptable levels of arsenic contents in all 16 water samples from school drinking water sources, indicating no significant threat to people’s health. This finding is in line with a study conducted in hilly parts of the Myagdi district, where values of arsenic are reported to be within the NDWQS [17]. Other studies conducted in Asia (e.g., Cambodia) showed higher levels of arsenic (0.13–0.2 µg/L) and lead (0.1–0.3 µg/L) in drinking water [45].

The high values of TTC are indicative of polluted drinking water sources or drinking water vessels, and of inadequate sanitary integrity of the water source and vessels [40]. Such contamination may be due to construction defects, poor sanitation, poor hand hygiene, and open defecation by freely roaming animals and humans in close proximity to open water sources [40]. In our study, the microbiological analyses of water samples revealed the presence of TTC in 193 water samples with 81(42.0%) of these samples having a TTC >100 CFU/100 mL, which calls for urgent treatment. Of note, despite households reporting that they obtain water from improved sources, faecal contamination was still observed in some of these. Yet, this water was being consumed by schoolchildren. Additionally, some improved water sources in the community were also not free from faecal contamination. This observation highlights that “improved” drinking water sources, considered safe by the global monitoring framework and burden of disease analyses, may entail health risks at some sources [21,46,47,48]. Cross-contamination at leakage points in old pipes, back siphoning, and drainage systems had been reported by a study conducted in Myagdi district and mountainous parts of Nepal [17,49]. Our findings of water contamination with TTC might be also linked to garbage discarded in open spaces in close proximity to drinking water points, open defecation practices, or cross-contamination between water supply and sewage systems.

The practice of open defecation was still common in the study region. Indeed, 17% of the households surveyed reported open defecation. However, this percentage of households practicing open defecation is considerably lower than what has been reported by WHO/UNICEF JMP in Nepal, where 37% of the rural population reported to practice open defecation [1,50]. This difference might be explained by the fact that temporary latrines were constructed immediately after the April 2015 earthquake with Dolakha district being the epicentre [51]. It should also be noted that some VDCs of the Dolakha district had declared the states of “open defecation-free”.

Re-growth of TTC in drinking water sources occurs at temperature above 15 °C, in the presence of sufficient bacterial nutrients and the absence of free residual chlorine in the water [44]. In our study area, some sites were located in settings where the average temperature is above 15 °C. Of note, we found that more than 85% of the households where TTC was present reported no treatment of drinking water, while only 13.5% reported treatment. Boiling was the most frequently known water treatment procedure; however, boiling alone might not confer full protection from TTC. The finding of boiling as the main known water treatment procedure is in line with a previous study conducted in rural Nepal, where 15% of households consistently boiled water before consumption [52,53]. Additionally, poor maintenance of sanitation facilities and inefficient disinfection are other likely reasons for the observed TTC contamination in our study.

In terms of KAP on WASH among schoolchildren, results indicated that 97% of the students reported washing their hands solely with water when soap was not available and 97.3% reported using soap and water for handwashing if soap was available. Of note, the presence of soap and water is crucial for schoolchildren for handwashing in that it might form a sustained habit of proper handwashing. Similar findings of the importance of the availability of soap and water were reported in studies conducted elsewhere such as in Nepal, Bangladesh, Nicaragua, and Kenya [39,53,54,55,56].

In terms of knowledge of water-borne diseases, children had a general awareness that dirty water can cause ill-health. Yet, the exact types of water-borne diseases and transmission pathways were poorly understood, thus confirming previous observations made in South Africa where the schoolchildren from rural schools were reported to have a disparity of knowledge on water-borne diseases [41]. It follows that the provision of adequate resources and long-term behaviour change in children to form a sustained habit of hygienic behaviours such as washing hands with soap, including awareness regarding water-borne disease with its mode of transmission, should be initiated in the VgtS study site of Nepal. There was a lack of access to sufficient quantities of water and soap at the unit of both school and households that impedes personal hygiene [56,57,58]. WHO recommends minimum availability of 100 L of water per capita per day for all purposes [59,60].

We found a significant association between the presence of TTC contamination of drinking water and domestic animals freely roaming within the households compared to households where domestic animals were kept outside. This might be due to faecal contamination of water sources by domestic animals. Such faecal contamination of drinking water and possession of different types of livestock was also reported in studies conducted in Burkina Faso and Rwanda [61,62,63]. We observed inadequate washing of drinking water storage containers, containers having no lids, or lids not fitting properly, and drinking water cups left on dirty grounds, as well as kitchens in close proximity to animal sheds. Similar observations have been made in a study conducted in Botswana, where the drinking water containers were kept without lids [64].

Our study has several limitations. First, there was a huge challenge posed by the April 2015 earthquake. Around 20% of households could not be visited due to frequent aftershocks and post-earthquake emergency crisis. A number of villages had been severely destroyed, and hence, it was not possible to obtain water samples from all the schoolchildren’s households. Second, water quality analysis was carried out during the spring season only, thus the observed results might not represent the drinking water quality over the whole year. Third, although having found standard residual free chlorine in some samples, TTC was high. This might be explained by the time required to destroy bacteria, which depends on the type of bacteria, but as we did not further isolate bacteria, we are unable to investigate this issue further. Fourth, the results from selected school, household, and community water sources in the two districts of Dolakha and Ramechhap may not be considered to be representative for other parts of Nepal. Fifth, the self-reporting of diarrhoeal episodes among children’s caregivers may not be accurate. However, this is the standard procedure.

Despite these limitations, our study provides a baseline for the status of WASH indicators at selected schools, households, and communities in two districts of Nepal. We rigorously assessed water quality, including physicochemical, microbiological, and heavy metal contents, using Delagua kit and flame atomic absorption spectrophotometer. Information about self-reported morbidities helped for timely referral of children to health care delivery centres. Meanwhile, the analytical approach taken (i.e., multivariate analysis) allowed for adjustments of potential confounders, such as educational attainment of caregivers, socioeconomic status, and regional differences.

## 5. Conclusions

We found that about one-third of water samples obtained from selected schools and households in two districts of Nepal were unsafe for drinking. The microbiological characteristics were critical for some samples, which indicates a public health risk. Although the physicochemical parameters of the water samples collected were within permissible limits, disinfection with chlorine prior to supply, as recommended by the NDWQS, is required to maintain water quality at the source. Regarding point-of-use, contamination of drinking vessels by domestic animals freely roaming inside the houses is a concern. Households’ drinking water was mostly from improved sources; however, regular monitoring of water quality in different seasons is recommended to generate evidence regarding water quality throughout the year.

Water source protection strategies (e.g., proper fencing of domestic animals, maintenance and proper disposal of human and animal faeces) should be promoted. When school budgets do not allow for WASH improvements at schools, parents and community organisations might provide resources to ensure healthy school environments. Regular inspections are required to identify causes of contamination and to determine the risk of future contamination. In turn, mitigation measures can be implemented, such as maintenance and operation of water supply systems by the school administration, household caregivers, and other community stakeholders. Additionally, engaging the communities to take responsibility for management of water sources may also be an appropriate strategy to improve the quality of community water sources. Promotion of hand washing with soap and safe disposal of faeces must be encouraged at the unit of the school. More emphasis should be placed on water treatment. Hygiene training programmes at schools should be incorporated into the school curriculum.

Our study gathered helpful baseline WASH information at school, household, and community levels. In order to create better conditions for the VgtS project, specifically nutritional and health-related objectives, the results will be useful to design and implement a complementary WASH intervention package for targeted schools. As the VgtS project in Nepal has involved the Ministry of Education as a key stakeholder, the model of interventions implemented in these pilot schools could be readily replicated nationwide. There is a need for ensuring safe WASH in schools, households, and the communities to improve children’s health and wellbeing. The study has therefore a potential to impact on the public health in the surveyed districts and schools, and also beyond.

## Figures and Tables

**Table 1:** Characteristics of study population in Dolakha and Ramechhap districts, Nepal, March–May 2015.

Characteristics		Overall *N* (%)	Dolakha *n* (%)	Ramechhap *n* (%)
**Sex of the schoolchildren (*n* = 708)**				
Girls		339 (47.9)	261 (47.0)	78 (51.0)
Boys		369 (52.1)	294 (53.0)	75 (49.0)
**Age of children (*n* = 708)**				
Age group 1 (8–12 years)		108 (15.2)	86 (15.5)	22 (14.4)
Age group 2 (13–16 years)		600 (84.8)	469 (84.5)	131 (85.6)
**Education level of children (*n* = 708)**				
Grade 6		333 (47.0)	258 (46.5)	75 (49.0)
Grade 7		375 (53.0)	297 (53.5)	78 (51.0)
**Educational attainment of caregivers (*n* = 562)**				
No formal schooling		210 (37.4)	174 (39.2)	36 (30.5)
Primary education		144 (25.6)	130 (29.3)	14 (11.9)
Secondary education		143 (25.4)	82 (18.5)	61 (51.7)
Higher education		65 (11.6)	58 (13.0)	7 (5.9)
**Age of caregivers (*n* = 562)**				
18–24 years		2 (0.4)	1 (0.2)	1 (0.9)
24–40 years		239 (42.5)	184 (41.4)	55 (46.6)
>40 years		321 (57.1)	259 (58.3)	62 (52.5)
**Ethnicity of caregivers (*n* = 562)**				
Brahmin		101 (17.9)	97 (21.9)	4 (3.4)
Chhetri		210 (37.4)	154 (34.7)	56 (47.5)
Newar		33 (5.9)	22 (4.9)	11 (9.3)
Tamang		213 (37.9)	166 (37.4)	47 (39.8)
Janajati		5 (0.9)	5 (1.1)	0 (0.0)
**Socioeconomic status of caregivers (*n* = 562)**				
Roof material	Corrugated iron roof	415 (73.8)	325 (73.2)	90 (76.3)
	Wood and tiles	147 (26.2)	119 (26.8)	28 (23.7)
Wall material	Wood	66 (11.7)	61 (13.7)	5 (4.2)
	Corrugated iron	407 (72.4)	331 (74.6)	76 (64.4)
	Bricks	89 (15.9)	52 (11.7)	37 (31.4)
Floor material	Mud	524 (93.2)	430 (96.9)	94 (79.7)
	Cement	38 (6.8)	14 (3.1)	24 (20.3)
Socioeconomic status * (*n* = 562)	High	49 (8.7)	39 (8.8)	10 (8.5)
	Middle	215 (38.3)	163 (36.7)	52 (44.1)
	Poor	298 (53.0)	242 (54.5)	56 (47.5)
**Owning agricultural land (*n* = 562)**		511 (90.9)	412 (92.8)	99 (83.9)
**Possession of domestic animals (*n* = 562)**		507 (90.2)	401 (90.3)	106 (89.8)

The mean (± SD) age of schoolchildren was 12.8 (±1.2) years. The median age of caregivers was 39.5 with an interquartile range of 11 years. * A new variable for socioeconomic status was created using factor analysis of 13 binary variables indicating the possession of household assets such as a radio, television, mobile phone, table, stove, petroleum lamp, gas lamp, etc. The score of the first factor was then classified into three wealth categories (high, middle, or poor) using the k-means procedure.

**Table 2:** School characteristics and WASH (water quality, sanitation, and hygiene) conditions in Dolakha and Ramechhap districts, Nepal, March–May 2015.

Variables		Overall *N* (%)	Dolakha *n* (%)	Ramechhap *n* (%)
**School level**				
Secondary		10 (62.5)	9 (69.2)	1 (33.3)
Above secondary		6 (37.5)	4 (30.8)	2 (66.7)
**School building age**				
0–10 years		7 (43.7)	6 (46.2)	1 (33.3)
11–20 years		4 (25.0)	3 (23.1)	1 (33.3)
>20 years		5 (31.2)	4 (30.8)	1 (33.4)
**School size**				
<500 students		4 (25.0)	3 (23.1)	1 (33.3)
>500 students		12 (75.0)	10 (76.9)	2 (66.7)
**School conditions**				
Electricity at the school		11 (68.7)	9 (69.2)	2 (18.2)
Roof material	Iron sheet	15 (93.7)	13 (100.0)	2 (66.7)
	Bamboo	1 (6.3)	0 (0.0)	1 (33.3)
Floor material	Cement	2 (12.5)	0 (0.0)	1 (33.3)
	Soil	14 (87.5)	12 (92.3)	2 (66.7)
Wall material	Brick	2 (12.5)	1 (7.7)	1 (33.3)
	Bamboo	2 (12.5)	0 (0.0)	2 (66.7)
	Iron sheet	12 (75.0)	12 (92.3)	0 (0.0)
Water source *				
Surface water		2 (12.5)	0 (0.0)	2 (66.7)
Standpipe		6 (37.5)	5 (38.5)	1 (33.3)
Piped water		8 (50.0)	8 (61.5)	0 (0.0)
Drinking water *				
Drinking water available throughout year		7 (43.8)	7 (100.0)	0 (0.0)
Treatment of drinking water by school		0 (0.0)	0 (0.0)	0 (0.0)
**Water in school ^#^**	Inadequate	14 (87.5)	13 (100.0)	1 (33.3)
	Adequate	2 (12.5)	0 (0.0)	2 (66.7)
**Hand washing facility in any area of school**		0 (0.0)	0 (0.0)	0 (0.0)
**Latrine**				
Flush toilet		3 (18.8)	2 (15.4)	1 (33.3)
Pit latrine with cement floor and composting latrine		4 (25.0)	4 (30.8)	0 (0.0)
Pit latrine without cement floors, hanging latrine		9 (56.2)	7 (53.9)	2 (66.8)
**Latrine condition**				
Presence of door		16 (100.0)	13 (100.0)	3 (100.0)
Sharing with opposite gender		14 (87.5)	12 (92.3)	2 (66.7)
Damaged floor ^(i)^		9 (56.3)	8 (61.5)	1 (33.3)
No privacy ^(ii)^		8 (50.0)	7 (53.9)	1 (33.3)
Clean floor ^(iii)^		5 (31.3)	4 (30.8)	1 (33.3)
Clean wall ^(iv)^		7 (43.8)	6 (46.2)	1 (33.3)
Flies present		8 (50.0)	5 (38.5)	3 (100.0)
Odour present		15 (93.8)	12 (92.3)	3 (100.0)
Regular water for anal cleansing		2 (12.5)	2 (15.4)	0 (0.0)
Washbasin for handwashing		0 (16.0)	0 (0.0)	0 (0.0)
Water for hand washing		6 (37.5)	4 (30.8)	2 (66.4)
Soap for hand washing		0 (0.0)	0 (0.0)	0 (0.0)
**Sanitation in school ^(v)^**	Inadequate	8 (50.0)	7 (53.9)	1 (33.3)
	Adequate	8 (50.0)	6 (46.1)	2 (66.7)
**Hygiene in school ^(vi)^**	Inadequate	8 (50.0)	6 (46.2)	2 (66.7)
	Adequate	8 (50.0)	7 (53.9)	1 (33.3)
**Safe solid waste disposal ^(vii)^**		0 (0.0)	0 (0.0)	0 (0.0)

* Multiple responses were possible for the variables. ^#^ A new variable for water adequacy/inadequacy was created using factor analysis with conceptually similar categorical variables of: types of water source in school (surface water, borehole/tube well/protected dug well, standpipe, rainwater, protected spring, unprotected dug well, piped water); number of these sources available (1–2, >2), drinking water availability during the day of survey (yes/no). Water quality was not considered for calculating water adequacy. ^(i)^ Floor is cracked, broken into separate pieces, fallen into the pit; ^(ii)^ walls with holes or no walls; ^(iii)^ presence of faeces, urine, dirt, ^(iv)^ presence of faeces on the wall; ^(v)^ a new variable for sanitation was created using factor analysis of variables characterising types of latrine (flush/pit latrine with cement floor and composting latrine and pit latrine without cement floors, hanging latrines, door, sharing by both sexes, damaged latrine floor, privacy, clean floor, clean wall, roof, flies, and odour); ^(vi)^ a new variable for hygiene was created using factor analysis of conceptually similar binary variables of: hygiene such as broom, regular water for anal cleansing, sanitary bins, water and soap, and washbasins for hand washing, solid waste disposal; and ^(vii)^ schools having reported to dispose their solid waste were expected through burial in safe place, collection at a safe place, or disposal. The score of the first factor was then classified into two categories adequate and inadequate, using the k-means procedure.

**Table 3:** Physicochemical and microbiological parameters of water samples in school and community in Dolakha and Ramechhap districts, Nepal (sampling period: May and June 2015).

Category	Parameter	Unit	Range	School						Community		
				Overall		Dolakha		Ramechhap		Overall	Dolakha	Ramechhap
				Main Source * (*N* = 16)	Point-of-Use ** (*N* = 13)	Main Source * (*n* = 13)	Point-of-Use (*n* = 10)	Main Source * (*n* = 3)	Point-of-Use (*n* = 3)	(*n*= 43)	(*n* = 33)	(*n* = 10)
				*n* (%)	*n* (%)	*n* (%)	*n* (%)	*n* (%)	*n* (%)	*n* (%)	*n* (%)	*n* (%)
Physical characteristics	Turbidity	NTU	>5	0 (0.0)	1 (7.7)	0 (0.0)	1 (10.0)	0 (0.0)	0 (0.0)	4 (9.3)	3 (9.1)	1 (10.0)
			2–5	16 (100.0)	12 (92.3)	13 (100.0)	9 (90.0)	3 (100.0)	3 (100.0)	39 (90.7)	30 (90.9)	9 (90.9)
	pH		6.5–8.5	0 (0.0)	13 (100.0)	13 (100.0)	10 (100.0)	3 (100.0)	3 (100.0)	43 (100.0)	33 (100.0)	10 (100.0)
Chemical characteristics	Free residual chlorine ***		0.1–0.2	16 (100.0)	13 (100.0)	13 (100.0)	10 (100.0)	3 (100.0)	3 (100.0)	43 (100.0)	33 (100.0)	10 (100.0)
	Total residual chlorine	mg/L	0.2–0.5	16 (100.0)	13 (100.0)	13 (100.0)	10 (100.0)	3 (100.0)	3 (100.0)	41 (95.3)	31 (93.9)	10 (100.0)
			0–0.199	0 (0.0)	0 (0.0)	0 (0.0)	0 (0.0)	0 (0.0)	0 (0.0)	2 (4.7)	2 (15.1)	0 (0.0)
	Lead	mg/L	<0.01	16 (100.0)	0 (0.0)	13 (100.0)	0 (0.0)	3 (100.0)	0 (0.0)			
	Arsenic	mg/L	<0.05	16 (100.0)	0 (0.0)	13 (100.0)	0 (0.0)	3 (100.0)	0 (0.0)			
Microbiological characteristics	Thermo-tolerant coliforms (TTC) ***	CFU/100 mL	<1	4 (25.0)	3 (23.1)	3 (23.1)	3 (30.0)	1 (33.3)	0 (0.0)	26 (60.5)	21 (63.6)	5 (50.0)
			1–10	5 (31.3)	4 (30.8)	5 (38.5)	2 (20.0)	0 (0.0)	2 (66.7)	3 (7.0)	2 (6.0)	1 (10.0)
			11–100	6 (37.5)	2 (15.4)	4 (30.8)	2 (20.0)	2 (66.7)	0 (0.0)	8 (18.6)	6 (18.2)	2 (20.0)
			>100	1 (6.2)	4 (30.7)	1 (7.6)	3 (30.0)	0 (0.0)	1 (33.3)	6 (13.9)	4 (12.1)	2 (20.0)

* Main sources are the main drinking water sources, such as stand pipes, piped water, and spring water, which are available at the school. ** Point-of-use is the drinking water cups used for drinking water by the surveyed school-aged children; *** The presence of TTC despite the residual chlorine at acceptable range depends on the “contact time” and the bacterial type. CFU, Colony forming unit; NTU, Nephelometric turbidity unit.

**Table 4:** Questionnaire findings on KAP ^(i)^ on WASH among schoolchildren and caregivers in Dolakha and Ramechhap districts, Nepal, March–May 2015.

Variables (*n* = 708)	Overall *N* (%)	Dolakha *n* (%)	Ramechhap *n* (%)
**KAP indicators: schoolchildren**			
Hand washing			
Before eating	525 (74.2)	427 (76.9)	98 (64.1)
After eating	434 (61.3)	357 (64.3)	77 (50.3)
After playing	422 (59.6)	345 (62.2)	77 (50.3)
After defecation	534 (75.4)	427 (76.9)	107 (69.9)
Do not wash hands	66 (11.7)	45 (10.1)	21 (17.8)
With water only	687 (97.0)	540 (97.3)	147 (96.1)
With ash	17 (2.4)	12 (2.2)	5 (3.3)
With mud/soil	4 (0.6)	4 (0.7)	0 (0.0)
With water and soap	689 (97.3)	539 (97.1)	150 (98.0)
**Hygiene ^(ii)^**			
Higher category	261 (36.9)	225 (40.5)	36 (23.5)
Middle category	211 (29.8)	165 (29.7)	46 (30.1)
Lower category	236 (33.3)	165 (29.7)	71 (46.4)
**Sanitary practices at school**			
Using latrine at school	679 (95.9)	543 (97.8)	136 (88.9)
No latrine use	29 (4.1)	12 (2.2)	17 (11.1)
Drinking water of children at school *			
Drinking water from school	637 (90.0)	535 (96.4)	102 (66.7)
Bringing water from home	102 (14.4)	67 (12.1)	35 (22.9)
**Households (*n* = 562)**			
Use of toilet at home			
Latrine in the household	394 (70.1)	320 (72.1)	74 (62.7)
Shared latrine	68 (12.1)	57 (12.8)	11 (9.3)
Bush	73 (13.0)	57 (12.8)	16 (13.5)
River, swamp, lake	27 (4.8)	10 (2.2)	17 (14.4)
**Type of latrine at home**			
Water seal latrine	283 (50.4)	233 (52.5)	50 (42.4)
Open pit latrine with slab	97 (17.3)	77 (17.3)	20 (16.9)
Open pit latrine without slab	14 (2.5)	12 (2.7)	2 (1.7)
Soap in household for hand-washing	417 (74.2)	319 (71.9)	98 (83.0)
**Hygiene of caregivers ^(iii)^ (*n* = 252)**			
Lower category	72 (28.7)	60 (27.0)	12 (41.4)
Middle category	26 (10.4)	23 (10.4)	3 (10.3)
Better category	153 (60.9)	139 (62.6)	14 (48.3)
Drinking water at home *			
*Drinking water source during dry season*			
Private tap	287 (51.1)	257 (57.9)	30 (25.4)
Spring	13 (2.3)	3 (0.7)	10 (8.5)
Public tap	36 (6.4)	36 (8.1)	0 (0.0)
Other ^(iv)^	226 (40.2)	148 (33.3)	78 (66.1)
*Drinking water source during rainy season*			
Private tap	285 (50.7)	258 (58.1)	27 (22.9)
Spring	1 (0.18)	1 (0.2)	0 (0.0)
Public tap	44 (7.8)	40 (9.0)	4 (3.4)
Other ^(v)^	232 (41.3)	145 (32.7)	87 (73.7)
**Container to fetch water at the principle source**			
Clay	40 (7.1)	16 (3.6)	24 (20.3)
Plastic	258 (45.9)	205 (46.2)	53 (44.9)
Metal	264 (47.0)	223 (50.2)	41 (34.8)
**Frequency of washing drinking water storage container with soap**			
Never	40 (7.1)	20 (4.5)	20 (17.0)
Daily	347 (61.8)	277 (62.4)	70 (59.3)
Weekly	175 (31.1)	147 (33.1)	28 (23.7)
**Status of drinking water container**			
Covered	417 (74.2)	322 (72.5)	95 (80.5)
Uncovered	145 (25.8)	122 (27.5)	23 (19.5)
**Drinking water container used for other activities**	112 (19.9)	89 (20.5)	23 (19.5)
Regular water treatment	76 (13.5)	50 (11.3)	26 (22.0)
Aware of boiling	203 (36.1)	181 (40.8)	22 (18.6)
Aware of chlorination	32 (5.7)	28 (6.3)	4 (3.4)
Aware of filtration	70 (12.5)	28 (6.3)	42 (35.6)
**Water sufficiency**	439 (78.1)	333 (75.0)	106 (89.8)
Safe solid waste disposal *	273 (48.6)	237 (53.4)	36 (30.5)

* Multiple answers were possible for several questions. ^(i)^ Knowledge, attitude, and practices; ^(ii)^ and ^(iii)^ a new variable for hygiene for the schoolchildren and their caregivers was created using factor analysis separately with two conceptually similar categorical variables of: mode of hand-washing (with water only, ash, mud/soil, water and soap, no hand washing); and its occasions (for schoolchildren: before eating, after eating, after playing, after toilet, and for their caregivers: before preparing food, before eating, after eating, after defecation, after child’s defecation, before breastfeeding, after breastfeeding, no hand-washing; the score of the first factor was then classified into three categories - high, middle, and low using the k-means procedure; ^(iv)^ and ^(v)^ others included hand-pump, river, swamp, and ponds.

**Table 5:** Physicochemical and bacteriological parameters of point-of-use water samples in households in Dolakha and Ramechhap districts, Nepal (sampling period: June 2015).

Category	Parameter	Unit	Range	Overall *N* (%)	Dolakha *n* (%)	Ramechhap *n* (%)
Physical characteristics	Turbidity	NTU *	>5	131 (23.3)	115 (25.9)	16 (13.6)
			2–5	431 (76.7)	329 (74.1)	102 (86.4)
	pH		6.5–8.5	562 (100.0)	444 (100.0)	118 (100.0)
Chemical characteristics	Free residual chlorine	mg/L	0.3–0.5	121 (21.5)	105 (23.6)	16 (13.6)
			0.1–0.2	441 (78.5)	339 (76.4)	102 (86.4)
	Total residual chlorine	mg/L	>0.5	2 (0.4)	1 (0.2)	1 (0.8)
			0.2–0.5	548 (97.5)	439 (98.9)	109 (92.4)
			0–0.199	12 (2.1)	4 (0.9)	8 (6.8)
Microbiological characteristics	Thermo-tolerant coliforms (TTC)	CFU/100 mL **	<1	408 (72.6)	333 (75.0)	75 (63.6)
			1–10	42 (7.5)	24 (5.4)	18 (15.3)
			11–100	42 (7.5)	36 (81.1)	6 (5.1)
			>100	70 (12.5)	51 (11.5)	19 (16.1)

* Nephelometric turbidity unit; ** Colony forming unit.

**Table 6:** Results from univariate and multivariate logistic regression analysis for thermo-tolerant coliforms (TTC) from water samples from households of Dolakha and Ramechhap districts, Nepal (sampling period: March–May 2015).

Risk Factor	*N* (%)	Univariate Analysis			Multivariate Analysis		
		OR	95% CI	*p*-Value	aOR	95% CI	*p*-Value
**District**							
Dolakha	444 (79.0)	1.00			1.00		
Ramechhap	118 (21.0)	1.79	1.02–3.13	0.04	2.25	1.16–4.34	0.02
**Education of the respondent**							
No formal education	210 (37.4)	1.00					
Primary education	144 (25.6)	1.21	0.74–1.99	0.44	1.26	0.76–2.07	0.37
Secondary education	143 (25.4)	0.75	0.43–1.32	0.34	0.73	0.41–1.30	0.29
Superior	65 (11.6)	0.76	0.36–1.61	0.47	0.92	0.43–1.95	0.82
**Socioeconomic status**							
Low	298 (53.0)	1.00					
Medium	215 (38.3)	1.10	0.73–1.64	0.65	1.07	0.71–1.61	0.75
High	49 (8.7)	1.06	0.53–2.11	0.87	1.02	0.50–2.06	0.97
**Household drinking water during the dry season**							
Private tap	287 (51.1)	1.00			1.00		
Spring	13 (2.3)	3.98	1.14–13.97	0.03	2.48	0.64–9.66	0.19
Public tap	36 (6.4)	1.67	0.70–3.95	0.24	1.68	0.71–3.96	0.23
Other	226 (40.2)	0.93	0.59–1.44	0.73	0.87	0.55–1.37	0.55
**Container for fetching water**							
Metal container	264 (47.0)	1.00					
Plastic container	258 (45.9)	1.11	0.73–1.69	0.62	0.96	0.60–1.52	0.85
Clay pot	40 (7.1)	1.86	0.86–4.02	0.12	0.82	0.34–1.99	0.67
**Status of container during storage**							
Covered	417 (74.2)	1.00					
Uncovered	127 (22.6)	0.97	0.60–1.57	0.89			
Not seen	18 (3.2)	0.90	0.30–2.70	0.86			
**Container washing frequency**							
Daily	347 (61.7)	1.00					
Never	40 (7.1)	0.82	0.35–1.93	0.65			
Weekly	175 (31.1)	0.95	0.60–1.51	0.82			
**Drinking water container used for other activities**							
Yes	451 (80.3)	1.00					
No	111 (19.7)	0.76	0.46–1.27	0.30			
Water treatment *							
No	486 (86.5)	1.00					
Yes	76 (13.5)	0.74	0.42–1.31	0.30			
**Latrine in household**							
Yes	395 (70.3)	1.00					
No	167 (29.7)	1.20	0.76–1.87	0.43			
**Latrine type**							
Water seal latrine	283 (50.4)	1.00					
No latrine	168 (29.9)	1.30	0.81–2.11	0.28			
Open pit latrine with slab	97 (17.3)	1.21	0.69–2.11	0.51			
Flushed toilet	14 (2.5)	0.91	0.24–3.49	0.89			
**Solid waste disposal**							
Yes	273 (48.6)	1.00					
No	289 (51.4)	0.99	0.67–1.48	0.99			
**Possession of livestock**							
Yes	507 (90.2)	1.00					
No	55 (9.8)	0.92	0.47–1.80	0.80			
**Livestock kept inside household**							
No	307 (54.6)	1.00					
Yes	255 (45.4)	0.63	0.41–0.95	0.03	1.64	1.08–2.50	0.02

* Households reported to treat their drinking water through boiling, chlorination, and filtration. The multivariate global model included a random intercept at the level of school where all the variables were assessed one by one and retained for the global model if their *p*-value was <0.2. The final model was obtained by using backward selection with the same level of <0.2.
